# Multiple pyonephrotic compartments with different microorganisms and an infected “calcium milk” in a patient with bilateral obstructive uropathy: A challenging case^☆^

**DOI:** 10.1016/j.ijscr.2022.107654

**Published:** 2022-09-13

**Authors:** Noor Buchholz, Anahita Dehghani, Hooman Kamran, Abdolreza Haghpanah

**Affiliations:** a U-merge Scientific Office, U-merge Ltd., London-Athens-Dubai, United Arab Emirates; b Laparoscopy Research Center, Shiraz University of Medical Sciences, Shiraz, Iran; c Student Research Committee, Shiraz University of Medical Sciences, Shiraz, Iran; d Endourology Ward, Department of Urology, Shiraz University of Medical Sciences, Shiraz, Iran

**Keywords:** Urolithiasis, Pyonephrosis, Kidney calculi, Urinary tract infections

## Abstract

**Introduction and importance:**

If the surgeon encounters frank pus, he is advised to limit the procedure to efficient drainage of the infected compartment of the urinary tract either by double J stent insertion or percutaneous nephrostomy and abort and postpone the definitive stone treatment until the infection is treated.

**Case presentation:**

We present a highly complex case of an elderly female with multiple obstructing stones in the left kidney and ureter, with complete staghorn stones in the right kidney. While this scenario was already complex by virtue of the stone burden alone, which demands the combination of multiple stone treatment techniques, it was further complicated by compartmental infections in various parts of the kidneys with different microbes necessitating repeated abortion of procedures. As often in elderly patients, there was no rise of inflammatory markers, and bladder urine cultures were repeatedly negative. Moreover, a rare form of infection was encountered, namely “calcium milk” in the form of a radio-opaque lower pole abscess on the right.

**Clinical discussion:**

We discuss the etiology, treatment, and management of pyonephrosis and remind the need to always take it into account and react accurately when encountering infected space behind obstruction during minimally invasive surgeries in urolithiasis.

**Conclusion:**

Hidden microorganisms with different entities should be considered during surgical management of urinary stones. Complete drainage and appropriate antibiotic therapy are the cornerstones of treating this condition.

## Highlights

• Hidden microorganisms with various entities should be considered for urinary stones.
• Obstructed parts of the urinary tract can form infected compartments in isolation.
• Complete drainage and antibiotic therapy are needed for treating pyonephrosis.
• It is important to stop the procedure and only apply drainage for purulent fluids.

## 1 Introduction

The importance of urine cultures before the intervention, during the intervention (if possible), and the culture of the stones themselves have been outlined in the literature [1]. If the surgeon encounters frank pus, he is advised to limit the procedure to efficient drainage of the infected compartment of the urinary tract either by double J (JJ) stent insertion or percutaneous nephrostomy and abort and postpone the definitive stone treatment until the infection is treated [2].

We intend to present a case of a 79-year-old female with a complex bilateral stones burden and ongoing infections in different compartments of the urinary tract with various microbial entities. In addition, “calcium milk”, a term for an infected radio-opaque calcium-containing fluid, mimicked a large portion of an almost complete staghorn stone in our case. Of note, the infectious agents were taken from the urine behind the stones, not from the stones themselves (not stone cultures). This case is not only complex in itself as defined by stone burden but rendered more complex by the above findings.

## 2 Case presentation

A 79-year-old female presented with a long-standing bilateral stone disease causing obstructive chronic kidney diseases (CKD) II. She had undergone previous percutaneous nephrolithotomy (PCNL) on the left side and suffered from controlled hypertension. The patient had mild bilateral flank pain with no history of fever. On presentation, serum creatinine was 1.81 mg/dl, and the estimated glomerular infiltration rate (eGFR) was 26 ml/min. Also, the inflammatory markers were in the normal range, and the bladder urine culture showed no growth.

**Fig. 1:**
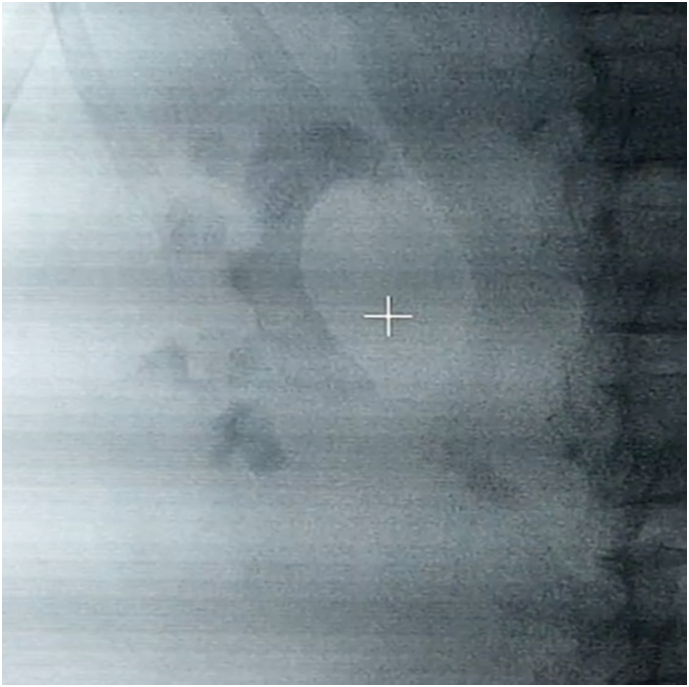
Non-contrast x-ray showed an almost complete staghorn stone in the right kidney and a sizeable upper ureter stone.

On the right side, there was an almost complete radio-opaque staghorn stone with a large branch filling most of the lower clubbed pole and two 1 cm stones, each one in the lower and middle poles, as well as a 1 cm stone in the upper ureter (Fig. 1). Besides, there was mild hydronephrosis. However, on the left side, there was a significant obstruction by two 1 cm stones in the upper and middle ureteral parts. In addition, in the middle pole of the left kidney, there were some residual stones from the previous PCNL. The patient wished for treatment as minimally invasive as possible. After discussing the advantages and limitations of each option with the patient, an initial uneventful extracorporeal shock wave lithotripsy (SWL) was performed on the more distal left ureteral stone.

After two weeks of follow-up, there were no radiological signs that the SWL had any fragmenting effect on the stone. The patient agreed to a ureteroscopy (URS) with laser stone fragmentation and insertion of a JJ stent. Pyonephrosis was encountered intraoperatively during URS. So, a culture was taken from the infected urine, a JJ stent was inserted, and the operation was aborted. Urine culture from the left renal pelvis showed *Pseudomonas aeruginosa* > 100,000 CFU/ml, sensitive to ciprofloxacin. So, ciprofloxacin was administered for ten days.

After one week, the urine culture was negative, and the patient was scheduled for another SWL of the ureteral stones with the JJ stent in situ. Only the more distal one was safely localizable and successfully fragmented in SWL.

**Fig. 2:**
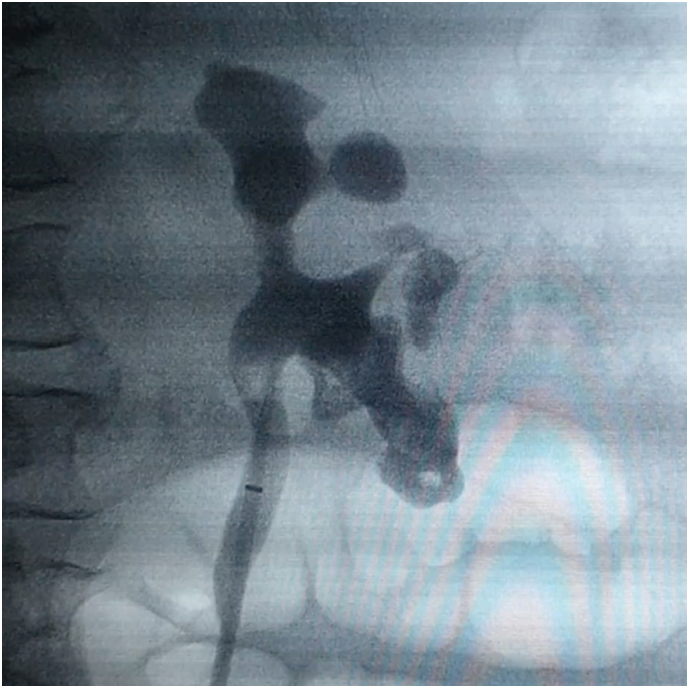
Matrix stones in the upper ureter, middle and lower pole of the left kidney impressing as solid stones on retrograde pyelography.

She was then scheduled two weeks later for URS to remove the remaining ureteral and middle pole stones. During the operation, it became clear that the stones in the left kidney were an agglomeration of small stones, with most of the material being calcified soft matrix (Fig. 2). Stones were fragmented completely, and matrix material with cloudy urine was drained as far as possible through a flexible ureteroscope by washouts through an access sheath. A JJ stent was also left behind. Urine culture from the urine behind the stone matrix showed *Escherichia coli* > 100,000 CFU/ml and *Streptococcus*, sensitive to amoxicillin/clavulanic acid and ciprofloxacin, respectively. Therefore, she was treated with both antibiotics for two weeks.

**Fig. 3:**
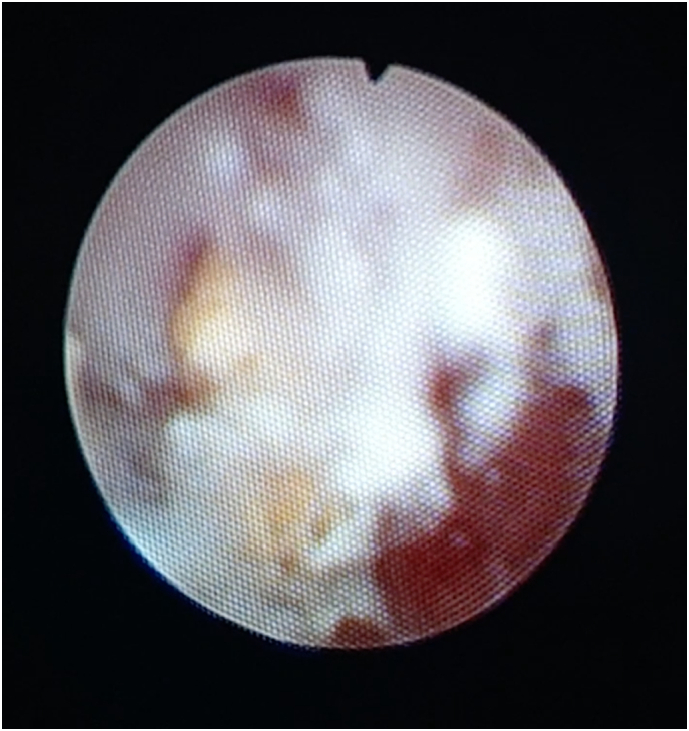
Endoscopic picture of stone matrix and in the background, stone fragments from the previous ureteroscopy and laser fragmentation in the left kidney.

A follow-up ultrasound three weeks later still reported stones of 1 cm each in the middle and lower poles of the left kidney. After a suitable recovery period of four weeks, the patient was scheduled for a further endoscopic second look in the left kidney and PCNL on the right side. On the left-sided intra-renal inspection, the solid stones previously seen in ultrasound were found to be an accumulation of small fragments in both poles, with further calcified matrix agglutinating them (Fig. 3). Further matrix was removed, and stones were re-fragmented to even finer gravel. A JJ stent was left in situ. In the same session, we intended to proceed to the right PCNL. The upper ureter stone was now completely obstructive and impacted. With some manipulation, we succeeded in advancing a ureteral catheter into the right kidney. Unfortunately, frank pus came out from hiding pyonephrosis behind the stone, and the operation was aborted once again at that stage. The right renal pelvic urine culture showed heavy growth of *extended-spectrum beta-lactamase (ESBL)-positive Escherichia coli* and *Proteus mirabilis.* According to sensitivity testing, the patient was treated with nitrofurantoin and ciprofloxacin for *Escherichia coli* and amoxicillin/clavulanic acid for *Proteus mirabilis*, respectively.

**Fig. 4:**
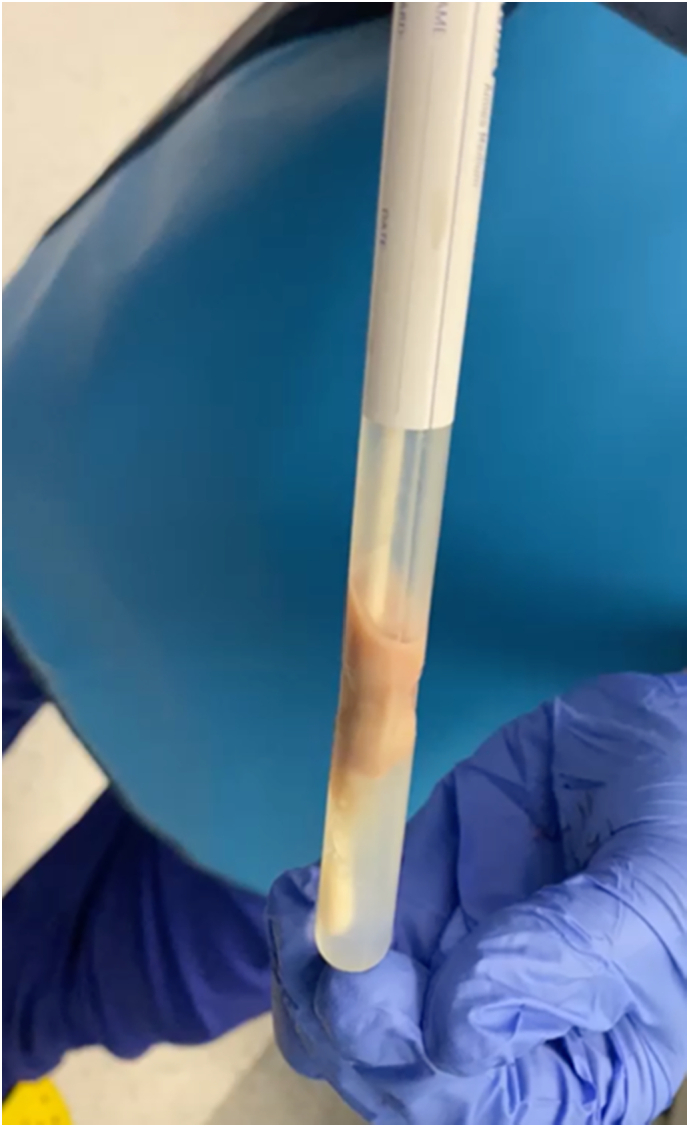
Purulent “calcium milk” aspirated from the lower pole of the right kidney.

**Fig. 5:**
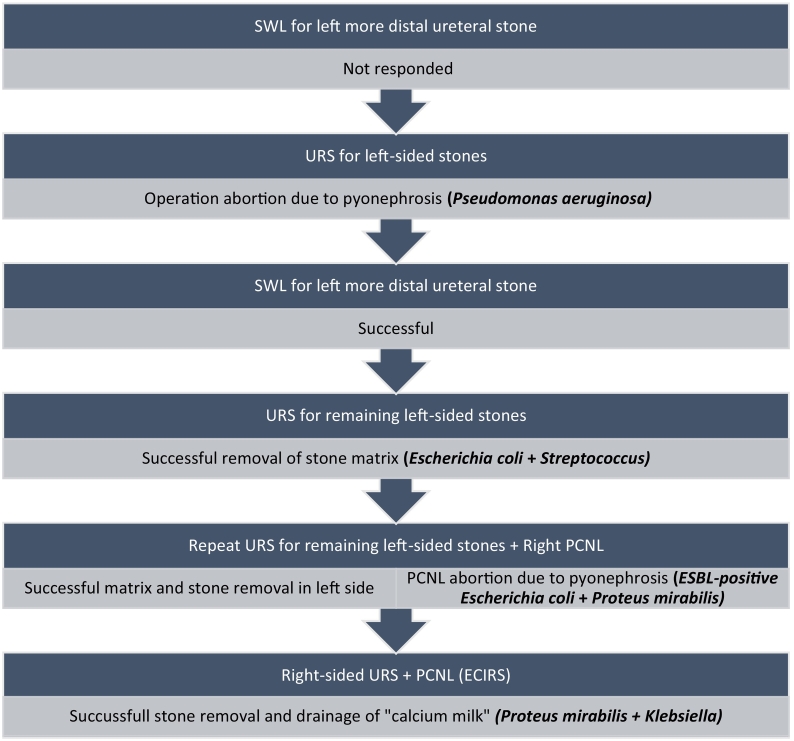
Management course of the patient; SWL: extracorporeal shock wave lithotripsy, URS: ureteroscopy, PCNL: percutaneous nephrolithotomy, ESBL: extended-spectrum beta-lactamase, ECIRS: endoscopic combined intra-renal surgery.

The patient was kept on ciprofloxacin and amoxicillin/clavulanic acid for two weeks and scheduled to remove the left JJ stent and right upper ureteral URS and PCNL (endoscopic combined intra-renal surgery, ECIRS) one month later. After the fragmentation of the upper ureteral stone, a supra-XII rib percutaneous access was made with balloon dilatation into the middle pole right without the use of contrast. Most of the stone burden could be removed from the kidney's renal pelvis and upper pole. Intraoperatively, another stone was seen to be blocking the lower pole and could not be reached through this access; the lower pole remained strongly radio-opaque. Therefore, a second puncture into the lower pole was performed with the Bull's eye technique [3], which drained a mixture of calcified milky fluid and pus (Fig. 4). Also, a nephrostomy tube was inserted and kept for two weeks. Culture revealed *Proteus mirabilis* and *Klebsiella* (80,000 CFU/ml) sensitive to aminoglycosides (adjusted to eGFR) and ciprofloxacin. After completing the course of antibiotics, the patient was discharged with a bilateral greatly reduced stone burden and an impaired but stable kidney function. Summarized treatment steps are demonstrated in Fig. 5.

The case has been reported in line with the SCARE 2020 criteria [4].

## 3 Discussion

Obstructed parts of the urinary tract can form infected compartments in isolation. It is well known that pyonephrosis, when isolated, may not even lead to tell-tale signs of severe infections, especially in the elderly. Since the infected compartment(s) do not directly communicate with the rest of the urinary system, bladder cultures may be negative and preoperative assessments may not indicate an ongoing infection. If, then, during the endoscopic intervention, the urologist encounters purulent fluids, the surgeon is strongly advised to drain the infected compartment using a JJ stent or nephrostomy tube to allow for decompression and to postpone the procedure until appropriate antibiotic therapy has been completed [2], [5].

In our case, over two months and several interventions, separate infections in such isolated compartments were encountered with negative bladder urine culture and normal inflammatory markers. These guidelines were respected every time, which of course, led to a significant delay in treatment. However, it cannot be emphasized enough how important it is to stop the procedure and only apply drainage despite cases reported where purulent fluid was encountered, and the procedure was completed without complications [2]. Also, our case shows that one may not rely on previous cultures taken from the contralateral or even the same kidney because one kidney may house more than one secluded obstructed compartment, which is infected with different microbes.

As in our case, the diagnosis of matrix stones is often only made during surgery. Complete clearance should be attempted, which is difficult with a retrograde approach. This is demonstrated by our case where ultrasound after the first left URS showed solid stones in the kidney, which turned out to be residual matrix and lasered fragments on the second look. More intriguingly, the lower pole on the right side of our patient posed as a solid lower part of a staghorn stone and, on the puncture, turned out to be purulent calcified “calcium milk”, as we termed it. We have not encountered this phenomenon of an intracalyceal abscess with such a high calcium concentration that it impressed as a stone on imaging before, nor have we found any references in the literature. Whether this was a liquefied matrix stone, heavily calcified pus, or a mixture of both remains speculation. Interestingly, culture from the ipsilateral kidney had shown *Proteus mirabilis* and *Escherichia coli*, commonly found in matrix stones [6]. The culture of the lower pole “calcium milk” revealed *Proteus mirabilis* again, but this time in combination with *Klebsiella*.

## 4 Conclusion

In conclusion, hidden microorganisms with different entities, which may lead to severe septic complications, should be considered during surgical management of urinary stones. Complete drainage and appropriate antibiotic therapy regarding culture are the cornerstones of treating this condition. Also, one should consider these hidden infections as one of the etiologies of unexplained postoperative fever in a patient with renal stones.

## Provenance and peer review

Not commissioned, externally peer-reviewed.

## Sources of funding

None.

## Ethical approval

N/A.

## Consent

Written informed consent was obtained from the patient for publication of this case report and accompanying images. A copy of the written consent is available for review by the Editor-in-Chief of this journal on request.

## Research registration

N/A.

## Guarantor

Abdolreza Haghpanah

## CRediT authorship contribution statement

N·B: study concept, data collection; A.D: writing the paper; H·K: revising the manuscript; A.H: study concept, revising the manuscript.

## Declaration of competing interest

None.
